# Just a small bunch of flowers: the botanical knowledge of students and the positive effects of courses in plant identification at German universities

**DOI:** 10.7717/peerj.6581

**Published:** 2019-03-13

**Authors:** Thorsten Buck, Ines Bruchmann, Pascale Zumstein, Claudia Drees

**Affiliations:** 1 Department of Sustainability Sciences, Institute for Ecology, Leuphana University Lüneburg, Lüneburg, Germany; 2 Ecology and Environmental Education, Institute for Biology and Chemistry, Universität Hildesheim, Germany; 3 Institute of Zoology, Universität Hamburg, Hamburg, Germany

**Keywords:** Biodiversity education, Expert knowledge, Conservation literacy, Higher education, Multiplier training

## Abstract

**Background:**

In the light of the ongoing loss of species the knowledge about and the ability to identify species becomes increasingly important for effective monitoring and conservation measures. Learning about identifying biodiversity is a central task for future biologists and biology teachers and universities play an important role in educating future experts and multipliers. It builds one basis for conservation literacy.

**Methods:**

We analyzed undergraduate students’ prior knowledge on plant species, identification and their knowledge gain from introductory plant identification courses at eight German universities. Using the Visual Classification Method—a combination of a presentation and standardized questionnaires—we evaluated the learning success of more than 500 students regarding (a) ‘declarative species knowledge’ of plant species names and (b) ‘taxonomic concept knowledge’, which is seen as knowledge on a higher level of complexity. From comparison of paired pre- and post-tests we calculated the individual knowledge gain. Using Linear Mixed Effects Models (LMMs) we analyzed effects of knowledge levels, learner-specific resources and learning environment on the knowledge gain.

**Results:**

We found that university course instructors have to start teaching at an almost zero level with respect to undergraduates’ prior knowledge: on average 2.6 of 32 common plant species were known. Overall, the introductory courses resulted in a significant but weak knowledge gain. We detected a higher knowledge gain in ‘taxonomic concept knowledge’ than in ‘declarative species knowledge’. We showed that the learning success was influenced by learner-specific resources, such as prior knowledge or aspects of motivation towards the subject matter, and by learning environment such as teaching methodology.

**Discussion:**

We discuss didactical demands and aspects of teaching methodologies that could facilitate learning the complex task of plant identification in university courses. Plant identification should be taught and supervised by experienced, highly motivated course instructors with profound expertise and outstanding didactical skills. In order to qualify future generations of biologists, biology teachers, or conservationists universities should aim at and encourage high-quality teaching.

## Introduction

The global loss of biodiversity has been identified as one of the major problems of global change (United Nations, 1992; Rockström et al., 2009; Newbold et al., 2016). Loss of biodiversity and ecosystem functions has severe ecological and socio-economic consequences (e.g., Oliver et al., 2015; Drescher et al., 2016) and results in a loss of both cultural and aesthetic values (Millennium Ecosystem Assessment, 2005).

Despite the increasing importance of conservation, society’s awareness for the need to take action is low (Miller, 2005; Kim & Byrne, 2006; Secretariat of the Convention on Biological Diversity, 2014). The ongoing loss of knowledge about biodiversity is problematic in terms of availability of future experts who will be able to describe and identify species or monitor long-term population trends (Miller, 2005; Kim & Byrne, 2006; Frobel & Schlumprecht, 2016). This increasing loss of knowledge raises concerns when it comes to training of multipliers in formal or non-formal education systems (as addressed in the Convention on Biological Diversity, United Nations, 1992; Trombulak et al., 2004).

Plant identification is one of the basic, yet complex, domains in biological sciences. Acquiring the basic skills of plant identification and thus simultaneously detecting, describing and learning about major concepts in biology such as taxonomy, diversity and variation or ecological dependencies, provides a basis for attaining higher levels of scientific understanding. The ability to determine species, however, can be considered not only as central competence of biological sciences (Uno, 2009) but also as an essential part for conservation literacy (Van Dijk, 2012; Smith et al., 2015). Institutes of higher education, especially universities, play a significant role in educating and training future biologists and taxonomists as well as future multipliers, such as teachers or environmental educators. In Germany, identification courses have seen significant cutbacks in terms of contents and intensity (Bromme et al., 2004). According to Bluethgen (2015), most universities still offer introductory identification courses, however, in almost one quarter of the biology programmes such courses are not compulsory anymore.

Several studies have revealed deficits in plant species knowledge among the young student or pupil generation (Hesse, 1984; Schussler & Olzak, 2008). An investigation by Bebbington (2005) in England found that the ability of students to identify and name common flowers is very limited and even trainee teachers and professional biology teachers who were tested performed unsatisfactorily. Moreover, most such studies only focus on what Bloom, Krathwohl & Masia (1956) call ‘declarative’ knowledge (i.e., the test persons’ ability to name single plant taxa, Anderson, Krathwohl & Bloom, 2001) but do not address knowledge gains at higher levels of complexity such as understanding of basic biological concepts or principles of (taxonomic) classification. Instead, we think that understanding of concepts such as the taxon plant *family* is crucial as it facilitates recognition and subsequent determination of a given plant *species*. To our knowledge, there is no study focussing on the assessment of learning success in plant introductory courses.

In our study we compared results from a set of pre-tests taken before and post-tests after participation in introductory plant identification courses. For the purpose of our study we categorized the knowledge gain in two pre-defined knowledge levels: (i) simple, fragmented, declarative knowledge of plant species names (henceforth ‘declarative species knowledge’) and (ii) the more complex, connected ‘taxonomic concept knowledge’, which includes a general understanding of the concept of taxonomic classification (Table 1). We analysed prior knowledge and learning success of undergraduate students using standardized questionnaires. We evaluated aspects of knowledge gain in ‘declarative species knowledge’ and ‘taxonomic concept knowledge’ of more than 500 students from eight German universities.

**Table 1:** Levels of knowledge differentiated in this study.

	**Level of knowledge**	
	**Declarative species knowledge**	**Taxonomic concept knowledge**
Level of complexity	Low	Moderate to high
Characteristics	Single terms, vocabulary fragmented, unconnected, simple often not contextualized and therefore easily forgotten	Understanding of a specific concept, more complex, integration of and connection between several knowledge elements, prerequisite for task-solving skills
Availability	Inert, not retrievable, cannot be accurately applied	Flexible
Level of competence(s) (Anderson, Krathwohl & Bloom, 2001)	*Remember*—retrieve relevant knowledge from memory, labelling	*Understand*—Construct meaning from instructional messages, including oral, written, and graphic communication
		*Analyse*—Break material into its constituent parts and determine how the parts relate to one another and to an overall structure or purpose
Level of cognitive skills (Crowe, Dirks & Wenderoth, 2008)	Lower-order cognitive skills: *knowledge*	Lower- and higher order cognitive skills: *comprehension* and *application*
Example in context of plant identification	Vocabulary for naming of single technical terms e.g., herb, gynoecium, carpel, decussate leaf arrangement Taxon names e.g., white dead-nettle (Lamiaceae)	The plant is an herb AND has a squared stem AND opposite, decussately arranged leaves AND bilaterally symmetric flowers reminiscent of an upper and lower lip, AND a gynoecium that shows two carpels (each carpel deeply lobed to mimic G4): This plant belongs to the Lamiaceae family.

We expect that (1) the students’ overall prior knowledge of species and plant systematics and taxonomy is generally low and that the students will enhance their knowledge while taking the introductory identification course. As plant identification courses mainly focus on teaching the concepts of plant systematics, such as plant families and their respective diagnostic features, (2) we presume a strong enhancement of students’ ‘taxonomic concept knowledge’ and only a moderate rise in ‘declarative species knowledge’ (i.e., ability to name species).

In order to deduce didactical or methodological demands that could facilitate coping with the complexity of a plant identification task and therefore promote successful teaching at universities, we also analysed potential factors influencing the learning processes in the courses. We expect that (3) learner-specific resources, such as prior knowledge, aspects of students’ motivation or interest towards the respective subject do positively influence the general learning success. Likewise, aspects of the learning environment (size of the learning group, study programme) and methodologies (whether or not a field trip was included) of teaching were analysed.

## Material and Methods

### Questionnaire and test procedure

**Figure 1:**
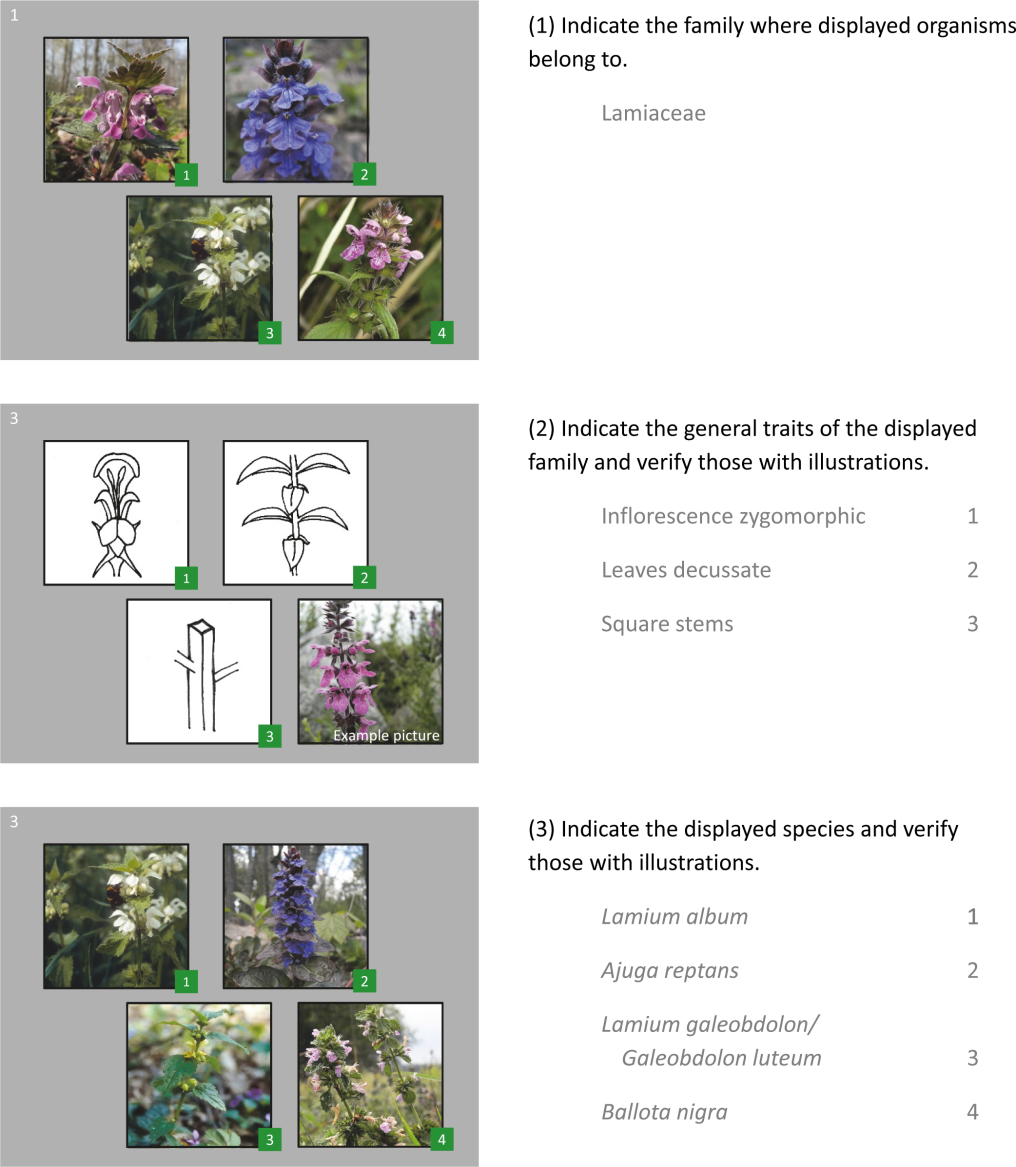
Example of a question cluster from the questionnaire for one plant family. Each cluster required the (1) identification of the plant family based on four pictures of common member species, (2) designation and assignment of three characteristic features for the identification of the respective plant family, and (3) identification and assignment of four species that are common members of the respective plant family. Example of a question cluster from the test showing questions (black) and correct answers and assignments (grey).

For evaluating the students’ knowledge gain in botanical taxonomy (categorized to the knowledge levels ‘declarative species knowledge’ and ‘taxonomic concept knowledge’, Table 1) we developed and used the ‘Visual Classification Method’. This method comprises standardized questionnaires with written questions combined with visual impressions presented in a simultaneous standardized presentation showing characteristic photographs and drawings (Fig. 1).

The questionnaire was based on open questions about the learning contents of beginners’ level plant identification courses queried from professional lecturers in botany. We interviewed course instructors about course contents and methods. We asked which plant families are regularly addressed and which representative species of these families are presented in the classroom. Of the 22 suggested plant families eight were chosen for the questionnaire (the most often named ones by instructors, except for the grass-like plant families). Course instructors were also asked about methodological aspects regarding their course design such as working with dichotomous keys, using fresh plant material for identification, herbaria work, as well as their planned modes of instruction (lecture—direct instruction; instructing seminar—interactive character; practical work on plant material; field trips—outdoor teaching). 14 instructors of eight universities took part in the study and gave comprehensive information about their planned course design and contents.

We grouped the learning contents in different tasks and structured the questionnaire as follows:

– identification and designation of the plant family based on four pictures of common member species (question 1),
– designation (question 2a) and assignment (question 2b) of three associated characters (=diagnostic features) relevant to the identification of the respective plant family (e.g., leaf arrangement, characteristics of inflorescence, leaves or seed capsules etc., based on simplified line drawings), and
– identification and designation (question 3a) and assignment (question 3b) of four species that are characteristic or representative members of the respective plant family (based on four pictures).

The questionnaire comprised eight common plant families (Asteraceae, Apiaceae, Brassicaceae, Caryophyllaceae, Fabaceae, Lamiaceae, Ranunculaceae, Rubiaceae) which include more than 30% of the floristic inventory of Germany (Federal Ministry for the Environment, 2014). The species list is given in Table S1. A pilot study to test the questionnaire was run in 2012 with 26 and 39 students at the Universities of Lüneburg and Flensburg, respectively. Experts further improved the questionnaire to exclude ambiguous plant photos or drawings of plant characteristics.

For the test of a possible influence of variables on learner-specific resources and learning environment on the score gain the questionnaire also contained questions to characterize the individual student, such as gender, age and the study programme in which a student was enrolled. Students estimated their percentage of attendance as well as time spent in self-study and reported whether they learned Latin at school or were a member of an environmental organization (for overview of variables and their justification see Table S2). We further collected data about course organization (with or without field trips, number of parallel classes, instructors). For reasons of privacy we were not able to exactly assign the students to their specific learning groups in which they had studied. The number of paired tests from the different learning group was taken instead as proxy for the ‘group size’. The amount of time spent on field trips was either zero (without field trip) or varied between six and 20 h per semester (with field trip). The questionnaires of both pre- and post-test are part of the Supplemental Information.

### Survey and scoring

Students from eight universities in Germany participated in this study. In order to avoid a possible bias due to the different school and higher education system in the former GDR we did not incorporate universities from former eastern Germany. General aspects of teaching methodology in the courses were comparable but varied between participating universities in terms of the extent of theoretical lectures, practical exercises, and supplementary field trips. All plant identification courses introduced the work with a dichotomous key (either Jäger, 2016; or Parolly et al., 2016). In all courses, freshly picked plants had to be identified.

In our study, we tested 1,060 undergraduate students in their second or fourth semester (study term) using our standardized questionnaire in the summer term 2013. The students were enrolled in the following biology-related study programmes: teaching with biology as the school subject, biology, or programmes in which elements of biology are taught, such as environmental sciences (for further information on participant numbers and return rates per university see Table S3; for information on distribution of the participants across study programmes, age structure, gender ratio of the participants or group sizes see Table S4). Each student was tested twice: at the beginning of the course (before the first lesson was given) (henceforth ‘pre-test’) and at the end of the course (henceforth ‘post-test’). The pre- and post-questionnaires were paired by an anonymous coding system for generating individual ID-codes allowing to measure the gain in knowledge individually. At the beginning of each test a pre-recorded standardized audio message provided necessary information on the purpose of the project and the test procedure. Learners were assured that the participation in the study was voluntarily and that results would not have any influence on their course grades. The respective course teachers approved our study by allowing us conducting the tests in their class rooms. Anonymity to both the participating students and teachers was likewise assured. The time frame for answering was standardized and visible to the test participants (the PowerPoint presentation changed slides automatically and remaining time was always displayed), the complete test lasted 24 min. During the tests, participants were observed to not copy any answers from other participants.

Using a predefined scoring sheet allowed objective and reliable scoring in a two-step-procedure: The correct naming of taxa or diagnostic features and the correct picture assignment were scored with one point each. Scoring was done standardized but generously according to a predefined list of scoring rules: both the full scientific and the regionally varying German common names were counted as correct (cf. Bebbington, 2005). Participants were not penalized for spelling mistakes. The identification of the correctly named plant genus scored half points.

The Ethics Committee of the Leuphana Universität Lüneburg granted ethical approval (no ethical concern) to carry out the study.

### The bunch of the best-known flowers

We analysed which of the 32 species (see Table S1) in the test (taken from question 3a) were best known by the participating students. For both pre-test and post-test, we counted how often a certain plant species was named correctly or semi-correctly.

### Statistical analyses

A gain in knowledge and identification ability (henceforth referred to as ‘score gain’) was evaluated by calculating the differences between pre- and post-test score for each test pair. For testing if the ability of naming corresponded to a correct assignment, the scores of questions 2a/b and 3a/b were correlated (Spearman rank correlation). For all other analyses only the results from questions 1, 2a and 3a were used.

Score gains were analysed using Linear Mixed Effects Models (LMMs) using the R package lme4 (Bates et al., 2015) (in R, v.3.0.2, R Development Core Team, 2013). Models were simplified in a backward selection procedure, in which each variable was taken out separately and compared (by a likelihood ratio test) to a model in which the respective variable was included. The variable that added the least amount of explanation was omitted from the model and the procedure started again. Only those variables were kept in the model which added a significant effect on the response variable (Crawley, 2007). As we were not interested in differences between universities and groups, we had ‘university/group’ and ‘university’ as (nested) random factors in the model. For the calculations on hypothesis 1 and 2 we accounted for pseudo-replication by also incorporating ‘ID’ into the random term (for further information see Table S2).

We defined that questions 1 and 2a needed ‘taxonomic concept knowledge’ for a correct answer, whereas question 3a only requires ‘declarative species knowledge’ (Table 1) and tested for differences in score gains between these two levels of knowledge gain and understanding. As we expected different results for the pre-test and post-test we tested for an interaction between the variables test-type and knowledge level.

For the test of a possible influence of learner-specific resources or learning environment on the score gain (hypothesis 3) the variables ‘scores pre-test’, ‘age’, ‘gender’, ‘Latin’, ‘organisation member’, ‘attendance’ and ‘self-study’ were included as fixed factors to the model regarding the learner-specific resources while the variables ‘group size’, ‘field trip’, ‘instructor’ and ‘study programme’ were added to the model on the learning environment (for further information see Table S2).

## Results

In total, 1,060 students from eight German universities took part in our study; 549 (51.8%) joined both test rounds (Table S3). A comparison of the pre-test scores among the participants without (*n* = 417) and with (*n* = 549) a paired post-test showed that the latter reached higher pre-test scores (LMM, *χ*^2^ = 12.87, *df* = 1, *p* < 0.001, Fig. S1). In the following, only the paired tests were analysed (*n* = 549). Most of the students tested were studying to become teachers of biology (47.9%), followed by 38.1% students studying biology, while remaining participants (14%) were enrolled in biology-related study programmes, such as environmental sciences (Table S4). 28.5% of the students were male, 35.2% had taken Latin at school, 8% were members of environmental organisations. The age of the students ranged from 17 to 35 with a median of 21 (Table S4).

The total number of tested students per university ranged from 57 (Frankfurt) to 280 (Gießen). We collected between 67 and 100% of the pre-tests and between 41 and 83% of the post-tests (for details see Table S3). Regarding the paired tests (pre-test and post-test from the same participant) the return rate ranged between universities from 22% to 88% (Table S3). Only 4.9% of the pre- and 0.2% of the post-tests had scores of 0; in none of the paired tests was the total score 0 in both tests. Thus, a refusal to cooperate on the part of some students can almost be ruled out.

### Learning success—comparison of pre- and post-tests

The post-test scores (mean 36.5 ± 15.3 standard deviation (SD) from a maximum of 120) were significantly higher than the pre-test scores (mean 8.1 ± 5.8 SD from 120) (LMM, *χ*^2^ = 1111.1, *df* = 1, *p* < 0.001, *n* = 549). The overall score gains in the tests ranged from −7 (i.e., a student had less knowledge in the post-test than in the pre-test) to 70, with a mean of 28.4 (±13.3 SD).

Results showed overall high correlations between questions on naming species (3a) or diagnostic features (2a) and the task of correctly assigning the displayed picture to the named species (3b) or feature (2b) (Spearman correlation coefficients >0.95 (*p* < 0.001) in all cases and tests, Table S5). All further analyses are based only on the tasks in questions 1 (plant family concept), 2a (diagnostic features), and 3a (naming species) for eight families (maximum score was 64).

### Learning species names

**Figure 2:**
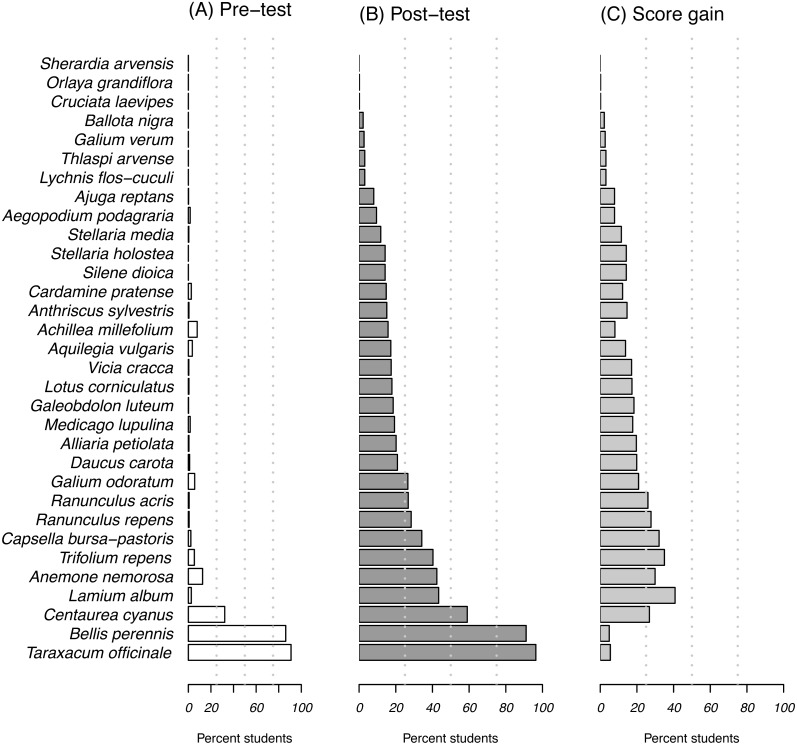
Abilities of the 549 students in the test to identify species. Bars indicate the percentage of correctly named species in pre-test (A) and post-test (B). The resulting score gain is shown in (C).

The students were able to identify and name the most ubiquitous plants dandelion (*Taraxacum officinale* agg., Asteraceae, 90.1%) and daisy (*Bellis perennis*, Asteraceae, 86.2%) before starting university studies. Of the 32 different species shown in the pre-test only four species were correctly identified by more than 10% of the students, whereas nine species were not recognized at all (Fig. 2, Table S1).

In the post-tests, 24 of the 32 species shown were identified and named correctly, with maximum percentages for the dandelion (96.4% of the students) and daisy (91.1%). The species with the highest gain in knowledge was white dead-nettle (*Lamium album*, Lamiaceae: 2.6% in pre- and 43.4% in post-tests). Only one plant species (blue field madder, *Sherardia arvensis*, Rubiaceae) was not correctly identified or named at all in either of the tests.

Plants from the Asteraceae family were the best-known in both pre- and post-tests by an average of 54.3% and 65.6% of students in the pre- and post-test, respectively. The least-known families were the Rubiaceae (on average 1.4%/7.4% of the students in pre- and post-test, respectively) and the Caryophyllaceae (0.1%/8.6%). The buttercup family (Ranunculaceae) was the family with the highest gain in scores, with the number of students identifying Ranunculaceae species increasing from 4.3% in the pre-test to 28.8% in the post-test; this was followed by the clover family (Fabaceae, increase from 1.6% to 22.0%). For all other tested plant families score gains ranged between 10% and 16%.

On average, a student named 2.6 (±8.7 SD) species correctly before taking the identification course, and identified 7.0 (±13 SD) of the 32 species in the post-test.

### Prior knowledge and knowledge gain

**Figure 3:**
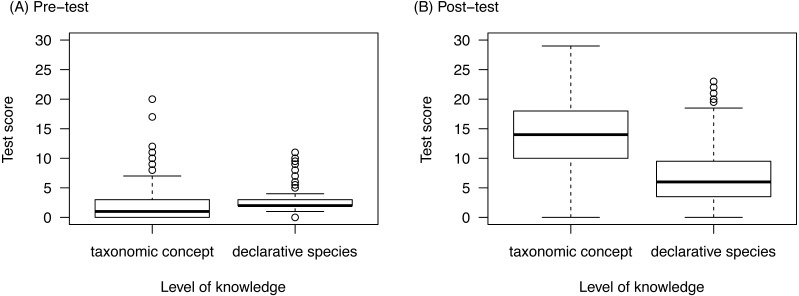
Differences in pre-test scores (prior knowledge, A) and post-test scores (B) for the different knowledge levels. Interaction between score-type and knowledge level: LMM, Chi^2^ = 652.1, *p* < 0.0001, df = 1, *n* = 549. The maximum score to be reached in the pre- and the post-test was 32.

**Figure 4:**
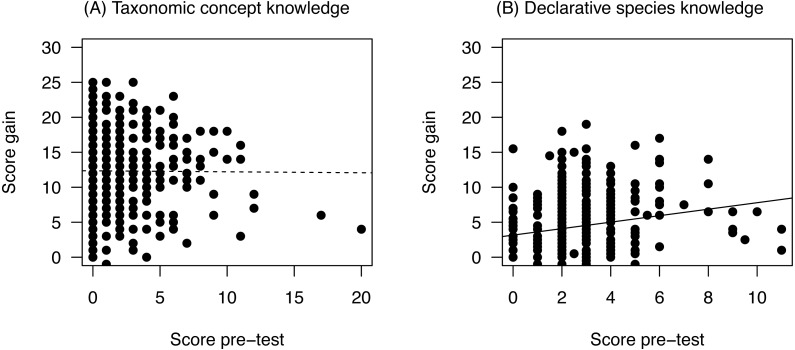
Relationship between score gain and pre-test scores according to knowledge level. Interaction between pre-test scores and knowledge level: LMM, Chi^2^ = 8.38, df = 1, *p* = 0.004, *n* = 549.

The pre-test scores were very low for both levels of knowledge: Students reached on average only 5.4% (±7.8% SD) in questions asking for ‘taxonomic concept knowledge’ and 8.1% (±4.8% SD) questions on ‘declarative species knowledge’. In the post-test we found a higher increase in ‘taxonomic concept knowledge’ than in ‘declarative species knowledge’ (Fig. 3). While the score gain for the ‘declarative species knowledge’ questions increased with pre-test scores (Fig. 4B) there was no relation to prior knowledge for the ‘taxonomic concept knowledge’ questions (Fig. 4A).

### Influence of learner-specific resources on the test results

The overall score gains differed depending on learner-specific characteristics. The higher the scores in the pre-test, the better the score gain (Table 2) (over questions 1, 2a and 3a). In general, females had higher score gains than male students (male students: 13.8, female students: 15.2; Table 2). Learners who had taken Latin at school did not perform better in the tests than those who had not. Likewise, membership in an environmental organization did not affect the test results (Table 2). While the influence of attendance was only marginally significant for the score gain, the amount of time spent in self-study had a positive effect on the test results (Table 2).

**Table 2:** Summary of test statistics from LMMs on effects of the learner’s investment on the overall score gain. Coefficients (coeff.) in brackets: coefficients of non-significant terms just before dropping the terms; other coefficients (not in brackets): from minimal adequate model (please note: coefficients in brackets cannot be compared to coefficients from the minimal adequate models, since the simplification alters coefficients); coefficients for a factor level (specified in brackets) give the difference to the reference level; bold *p*-values denote significant effects.

**Fixed effect**	**Coeff.**	**Chi^2^**	**DF**	***P***
(Mean)	13.813			
Scores pre-test	+0.305	10.70	1	**0.001**
Age	(−0.142)	2.26	1	0.133
Gender (female)	+1.357	4.46	1	**0.034**
Latin (no)	(−0.662)	1.24	1	0.266
Organisation member (no)	(−1.025)	0.88	1	0.348
Attendance (>50%)	(+2.668)	3.32	1	0.068
Self-study (more than 1 h a week)	+2.564	16.13	1	**<0.001**
**Random terms**	**Variance**			
University/group	6.05			
University	<0.001			
Residuals	40.26			

### Influence of learning environment on the test results

Learning environment variables had a significant impact on the students’ score gains. The main positive influence on score gains was whether parts of the course were taught outside of a classroom (i.e., field trip): students who were taught in a course including field trips reached on average 18.1, while students in courses without field trips collected on average only 5.9 score gain (Table 3). The study programme in which the students were enrolled and the instructing teaching person (course instructor) also had significant effects (Table 3). The size of the study group ranged from 17 to 65 and we found a negative trend towards an influence on the learning success, i.e., smaller groups tended to learn better.

**Table 3:** Summary of test statistics from LMMs on effects of the learning environment on the overall score gain. Coefficients (coeff.) in brackets: coefficients of non-significant terms just before dropping the terms; other coefficients (not in brackets): from minimal adequate model (please note: coefficients in brackets cannot be compared to coefficients from the minimal adequate models, since the simplification alters coefficients); coefficients for a factor level (specified in brackets) give the difference to the reference level; bold *p*-values denote significant effects.

**Fixed effect**	**Coeff.**	**DF**	**Chi^2^**	***P***
(Mean)	18.111			
Group size	(−0.163)	1	2.95	0.085
Field trip (no)	–12.159	1	58.51	**<0.001**
Instructor	+/ −^a^	13	56.76	**<0.001**
Study programme (teaching biology)	+2.314	2	6.00	**0.049**
Study programme (biology)	+0.566			
**Random term**	**Variance**			
University	0.00			
Residuals	42.9			

**Notes.**

a Coefficients are not given for reasons of privacy protection. Moreover, differences between the different instructors are beyond the scope of this work.

## Discussion

In our study we tested students from eight German universities enrolled in several biology-related study programs on their knowledge in plant identification before and after they had taken an introductory course. The students’ overall knowledge increased from 6.7% of the possible score in the pre-test to 30.4% in the post-test, with an average gain of 23.7%. The score gain was mostly due to a rise in ‘taxonomic concept knowledge’ (i.e., identifying the higher taxonomic rank ‘plant family’) rather than ‘declarative species knowledge’ (naming species). An increase in ‘declarative species knowledge’ (Table 1) depended on the pre-test scores, i.e., the more species a student knew prior to the course the more a student learned. The overall score gain depended on learner-specific characteristics, such as gender and the amount of time spent in self-study. Moreover, the score gain was influenced by the learning environment, with differences related to the instructor, the study programme a student had chosen and whether an identification course included a field trip.

### Students’ prior knowledge

We showed that students enter university with a poor overall knowledge of plant taxonomy and identification: On average, students barely reached 7% of possible scores in the pre-test. Thus, university instructors have to gear their teaching to the very low prior knowledge of students.

Students’ prior knowledge mainly consisted of ‘declarative species knowledge’ of ubiquitous species in the Asteraceae family, i.e., dandelion or daisy. At the taxonomic level of the plant family or its characteristics there was almost no knowledge present. Even though the students were allowed to answer with common plant names, the students’ knowledge of species names reached on average 2.6 species in the pre-test. Only few students performed better, with eleven (of 32 presented) correctly named plants. The prior knowledge of almost 80% of students, however, was restricted to three or fewer of the presented plant species. These results are in line with previous findings. Hesse (1984) and Lehnert (1999) both showed that undergraduates could only name five to six plant names (genus level only) when asked to free-list ten native herbal plants. Comparable to our study, dandelion and daisy were well known plant species.

Quantitative research on botanical knowledge at university level is scarce. Most studies on botanical knowledge have been conducted with pupils (e.g., Balmford et al., 2002; Lindemann-Matthies, 2006; Cooper, 2008). Bebbington (2005) showed that only 14% of A-level biology (school) students were able to name three or more common herbal wild plants of Britain. Lückmann & Menzel (2014) reported poor knowledge among German pupils (13–14 years) who, on average, named 2.3 herbal and 3.4 tree species (from eight species of each type).

Irrespective of the different target groups and applied testing approaches all studies show that children and young adults seem to have only poor floristic knowledge or ability to identify plants. One possible reason for the students’ poor prior knowledge might be an inherent disinterest in plants (Uno, 2009), called ‘plant blindness’ (Wandersee & Schussler, 2001; Balas & Momsen, 2014). Secondly, school-teaching seems to neglect botanical topics and therefore misses the chance of awakening interest in plants among pupils (Hershey, 1996; Lindemann-Matthies, 2005; Schussler & Olzak, 2008). Indeed, Hesse’s (2000) retrospective interview study revealed that most of the former pupils disliked learning about plants in school biology lessons. However, at the same time they also indicated that they missed the ability to name common species later on, in their adult life (Hesse, 2000). Most interviewed persons declared that their current species knowledge had been acquired more by informal (e.g., in family context) than by formal learning (i.e., at school).

Patrick & Tunnicliffe (2011) hypothesized that children show an innate interest in plants which, however, changes with age. Most adults do not notice or value botanical knowledge to be important to their personal life (Schneekloth, 1989). In a comparative study, Balmford et al. (2002) asked UK children about their knowledge of biological diversity (common UK plant and animal species) and artificial *Pokémon species* diversity. Eight-year-old children were substantially better at identifying Pokémon species than natural organisms such as a badger (*Meles meles*) or an oak (*Quercus* spec.). As the poor knowledge of natural species could not be ascribed to a reduced cognitive capacity of children to learn about species’ characteristics and names, the authors concluded that formal education in school often lacks inspiration in teaching about natural diversity and therefore misses important chances to enhance the children’s interest in learning about this topic.

### Learning success and gained knowledge of students

The overall knowledge gain of the students after one semester of plant identification course was low to moderate: The students attained on average only about 30% of total scores in the post-test and were at the end of semester able to identify and name, on average, 22% of the presented test species. A specific learning effect induced by the pre-test questionnaire probably influenced these results positively, as pre- and post-test questionnaires were identical. Thus, the true knowledge gain after the course might have been even lower. A systematic error due to the repeated test can be ruled out as the range of knowledge gain varied among the participants, and some participants showed no score gain at all. Moreover, we only analyzed those participants who had handed in both, a pre- and a post-test and this group performed significantly better than those students with only a pre-test. Thus, our results may be biased towards the more enthusiastic students. In summary, our results showed low prior knowledge of students and a positive but weak effect of introductory courses in plant identification on students’ gained knowledge.

To our knowledge, there are no comparable quantitative studies that evaluate floristic learning success in a highly standardized pre-post procedure. Most pre-post studies on species knowledge deal with animals (e.g., Randler, 2002; Randler, 2008), effects of varied teaching instructions on learning success (e.g., Goller, 2001; Lindemann-Matthies, 2006), or are based on small sample sizes (e.g., Fančovičová & Prokop, 2011; Stagg & Donkin, 2013). No study evaluates the target group of undergraduates in university training (i.e., at the level of tertiary education), thus the present study is the first to analyze this target group in a systematic way.

For teaching at university level the quality of gained knowledge (i.e., the degree of expertise) should be of special interest. According to Bromme et al. (2004), expert knowledge is described as acquisition and use of robust and flexible knowledge, which means that beginners in plant identification need to acquire dually-coded mental representations of diagnostic plant characteristics. On the one hand, they need to have a mental representation of significant features of the (perfectly grown) prototypic plant, and on the other hand students need to have mental representations of many possible variations of these diagnostic features (e.g., young *vs.* fully-grown plant individuals or growth forms of plants under shady or sunny conditions, etc.). Unfortunately, our data do not enable us to determine whether the knowledge gained by a student is only of rudimentary or fragmented quality (i.e., inert knowledge) or if a student reached a higher level allowing them to identify not only the optimally grown, flowering plant species (as in the illustrated pictures in the tests) but also specimens of the respective species in the field. Specifically designed tests should focus on this question in the future.

Regarding our results on learning success it remains a moot question whether one single plant identification course (generally 12–15 lessons of 120–180 min) can raise the students’ poor prior knowledge to the required degree of expertise in plant identification. Advanced courses in plant identification should be offered to further train interested students. Participation in guided plant mapping or field-work projects will help consolidate a student’s knowledge and expand their diagnostic abilities in the field (Dangerfield & Pik, 1999; Armbruster et al., 2009; Uno, 2009). Unfortunately, many universities in Germany act in a contradictory way e.g., by reducing contact hours in class or even squeezing plant and animal identification into one basic course (Bluethgen, 2015). In the face of the educational mission of universities to qualify people to become professionals in biology, teaching biology (didactics), and conservation, this trend is not promising.

### Learning contents and levels of knowledge

We showed that course instructors successfully conveyed ‘taxonomic concept knowledge’ to their students in introductory courses. This finding can be explained by (a) the set of general learning goals in introductory plant identification courses at university and (b) considering the *cognitive process* students have to take for achieving a progress in learning (Bransford, Brown & Cocking, 2000; Bromme et al., 2004).

The introductory plant identification courses that the participants of our study visited follow general learning goals: (i) differentiation between intra- and interspecific variation, (ii) knowledge of scientific rules for classifying (systematic ranking) and naming species, i.e., taxonomy and nomenclature, (iii) utilization of plant identification keys (i.e., ‘how-to’), and (iv) increasing the students’ knowledge on plant biodiversity and common or socio-economically important species.

It is not aimed that students learn about all 3,000 species of vascular plants known from Germany (Wisskirchen & Haeupler, 1998) in an introductory identification course. Instead, it is stated in the curricula and module handbooks that students should know the locally most common species when graduating their studies. Furthermore, students should become competent in scientific methods of identification and to be able to broaden their identification skills and species knowledge independently and professionally.

The subject-immanent complexity is demanding for both the learner as well the teacher (Armbruster et al., 2009; Lieu et al., 2017). For most of the above-named learning goals learners have to deal with knowledge at a higher level of complexity (concepts) than the task of simply memorizing terms and species names (Bromme et al., 2004). In our study every course instructor organized their teaching along the taxon level ‘plant family’ as central teaching content. With that instructors provide not only the relevant knowledge on taxonomy and systematic ordering of biodiversity in biological sciences to the learners but also offer a framework to the learners how to organise and interlink elements of knowledge in the cognitive learning process.

In our study the observed learning success was clearly gained in questions asking for plant families or their diagnostic features. An internalized plant family concept allows assigning plant species to certain plant families (or even genera) by simply checking the presence or absence of a set of diagnostic features. In applying their knowledge learners simultaneously build a link between theoretical scientific understanding and the practical task of plant identification; this leads to robust knowledge.

### Learners, instructors and learning success

Learning success in plant identification was positively correlated with the learner’s personal investment such as attendance in class or time spent in self-learning activities. Our study reveals that learners who invested only a minimum amount of time to the subject matter (one hour per week or less) did poorly in the test while students who declared to spent more than 1 h a week in learning and training reached significantly better results. The same is true regarding the time of attendance in class. Consequently, the motivation of a learner to deal with the subject matter and its specific challenges is essential to initiate successful learning processes. These findings imply the importance of a highly motivated and methodologically well-trained course instructor who is able to design a learning-friendly environment in class (Bryan, Glynn & Kittleson, 2011; Schunk & Zimmerman, 2012).

The contact time with course instructors varied between courses from 12 to 15 lessons of 120–180 min each. Thus, students only have little time to learn the large amount of scientific terms and concepts and to enhance their identification skills under supervision of a qualified course instructor. In order to offer to the students the chance to deal more intensively with the subject matter the learning schedule should offer additional phases of self-learning activities such as the visit of tutorials for identification exercises or the compilation of a herbarium as prerequisite for passing the exam. From a didactical point of view, these integrated phases of self-learning support the repetition and internalization of scientific terms, concepts as well as the transfer of the theoretical knowledge to the practical task of plant identification. Further, students get the chance to evaluate their learning progress, i.e., to realize learning contents they did not understand. A desirable result of frequent phases of self-learning would be that students prepare themselves for upcoming lessons in class which would enhance the individual learning progress (Lieu et al., 2017).

Our results indicate the tendency that students who attend the class regularly (more than 50%) showed higher scores in the test. Students who did not spend a reasonable time for exercising plant identification under supervision of a skilled instructor may suffer from lack of methodical skills (using a dichotomous identification key) or could easily get lost and frustrated because of undetected misconceptions (e.g., confusion of the terms ‘leaf’ and ,leaflet’). To facilitate learning and to overcome subject immanent learning barriers students should be encouraged to attend class frequently e.g., for exercising plant identification with freshly collected plant material under supervision of the course instructor. Qualified guidance and motivating feedback on the individual learning progress could positively influence a student’s self-efficacy and their general interest in plant sciences.

The influence of learner’s attendance in class and their investment on self-learning was as important as the learning environment. Thus, the mode of instruction is another prerequisite for successful learning in which a learner-centered atmosphere of support and guidance are of special importance: Clear instructions on tasks and learning goals within the learning arrangement were identified to be crucial to activate the learners’ cognition processes (Hattie, 2009). Transferred to our study, the central responsibilities of the course instructor teaching plant identification are (i) designing the learning environment, i.e., choosing teaching methods (mode of teaching, keys for determination, or plant specimens); (ii) anticipating subject-immanent learning barriers (e.g., numerous scientific terms, scientific nomenclature and basic concepts of taxonomic ordering) and diagnosing students’ possible misconceptions (e.g., if students confuse leaves and leaflets); (iii) providing expert knowledge, qualified guidance and feedback to enhance students’ learning progress, and (iv) promoting and motivating students’ self-efficacy (Bandura, 1997; Bandura, 2001) to master the task of successful plant identification (Armbruster et al., 2009; Schunk & Zimmerman, 2012).

All participating instructors decided to teach the identification task with dichotomous identification keys as these identification method gives special attention to details in plants morphology (similarity or dissimilarity). The anticipation or diagnosis of learner’s misconceptions is of special importance for a successful learning process (cf. Duit et al., 2012). Novice botanists not only have to internalize numerous botanical terms or concepts, they also have to learn *how* to apply their knowledge correctly, i.e., when working with dichotomous identification keys (which are for the most part based on text and line drawings). As described by Affeldt, Groß& Gropengießer (2010) and Affeldt, Groß& Stahl (2011) learners may be unable to apply dichotomous keys successfully because of pre-conceptions which are not in line with the scientific meaning of terms or concepts. Own observations and statements of interviewed colleagues teaching plant identification attest that many students confuse or misinterpret diagnostic relevant terms such as leaf and leaflets or flower and floral head (Asteraceae) and therefore fail an identification task. This can be overcome by the development of learner-friendly learning material which should incorporate an analysis of differences between scientists’ and students’ perspectives (thereby applying the Educational Reconstruction Model) (Affeldt, Groß& Gropengießer, 2010; Affeldt, Groß& Stahl, 2011). Only didactically well-trained instructors can guide students from the novice level to an advanced multidimensional level of botanical literacy and biological understanding (Baumert & Kunter, 2006; Van Dijk & Kattmann, 2007; Armbruster et al., 2009; Uno, 2009). In this context also group size is of importance as in smaller groups teachers have more time to guide a student in their learning process.

In our study we found a positive effect of the teaching method ‘field trip’ on learning success, despite varying intensities of realization of field trips. Field trips can be seen as experience-based learning arrangements (Rickingson et al., 2004). Here, a learner gets the opportunity to make guided first-hand experiences, i.e., to observe a plant species and its intraspecific variation in the natural habitat. Ideas on the ecological requirements of the species (wet, dry, shady, or sunny habitat) or aspects of plant sociology can be studied and discussed in the learning group.

A didactical advantage of first-hand experiences during outdoor learning is that learners have different ways of recognizing and integrating new information. When teaching in the field, course instructors could attract students’ interest in plants and taxonomy by contextualizing the relevance of knowledge with aspects of traditional uses or socio-economy (cf. Starosta, 1991; Goller, 2001; Kim et al., 2017). Learning additional facts about the species biology (e.g., species interactions, pollination, or spreading of species) may also help to interlink loose knowledge fragments. Moreover, outdoor learning arrangements promote students’ feelings to the relevance of the subject matter, or even their level of personal involvement, e.g., learning about threats on biodiversity and taking responsibility in conservation. In general, short field trips and, to an even greater degree, fieldwork projects have a high potential of raising students’ learning success (Dangerfield & Pik, 1999; Lindemann-Matthies, 2006; Fančovičová & Prokop, 2011; Stagg & Donkin, 2013).

Plant identification is a demanding and complex task. In order to acquire the skill of plant identification, beginners in botany have to spend a long time with learning, training and repeating to gain a sufficient degree of expertise (Bromme et al., 2004). Introductory courses constitute an important basis for the students’ understanding of basic concepts in taxonomic ordering and for the students’ ability to apply scientific methods in species identification. Ideally, instructors of introductory courses succeed to (spark the fire and) arouse a long-lasting interest in students to learn more about plant diversity, to improve their knowledge and abilities in specialized courses. Only if this basic groundwork was successfully established universities can qualify future experts.

## Conclusion and Recommendations

Our study revealed that the ‘Visual Classification Method’ is a suitable tool for assessment of learning success in introductory plant identification courses. Using this tool, we were able to differentiate between the two levels of knowledge ‘declarative species knowledge’ and the more complex ‘taxonomic concept knowledge’. We showed that ‘taxonomic concept knowledge’, in particular, was addressed in university teaching and that course instructors successfully conveyed the basic concepts to their students.

Introductory courses are only the first step in the training of professionals in biology, field ecology or teaching biology. Specialized advanced courses are advised to upgrade students’ knowledge and to broaden their skills towards their profession. In well-designed advanced courses the knowledge from the basic courses would be extended, more difficult taxa with their natural variation could be addressed. By adding more practical work and offering repeated cycles of learning under the supervision of an experienced course instructor, the knowledge would become robust and would reach the quality needed for successful and reliable identification of a plant. We recommend that smaller learning groups should be established where feedback and verification can be given more frequently by the course instructor, and misconceptions could be identified early allowing the learners to internalize the right concepts. In order to educate specialists universities should enrich the curricula of study programmes and (re-)integrate multiple courses on advanced topics to raise the student’s interest in species identification and knowledge.

Students can improve their knowledge and their skills in plant identification by self-learning activities. A teaching instruction integrating phases of self-learning activities (e.g., the compilation of a herbarium as examination requirement), will motivate students to deal more intensively with the subject matter. These self-learning phases can be enhanced if tutors with at a least moderate levels of identification skills give some guidance and feedback to the learners when needed. The offer of regular short field trips would motivate students to apply (i.e., to repeat and internalize) their knowledge. Moreover, field trips allow the students to get a set of first-hand-experiences.

As with other teaching, for introductory identification courses in botany or zoology, course instructors with profound expertise and outstanding didactical skills are required. Universities should encourage and reward course instructors that acquire both profound botanical or zoological expertise and outstanding didactical skills. When such instructors can include also other ways of teaching, such additional field trips for introductory plant identification courses, teaching can achieve more, and the gained levels of knowledge will rise. Only when this combination is given, will there be future generations of biologists or biology teachers who are able to identify species for ecological or conservation studies or to classify and describe plants or animals as species new to science.

##  Supplemental Information

10.7717/peerj.6581/supp-1 Figure S1 Comparison of pre-test scores of participants without and with paired post-test The maximum score to be reached was 64. LMM, *χ*^2^ = 12.87, *df* = 1, *p* < 0.001. Note, that this comparison is based on all questions of the questionnaire.

10.7717/peerj.6581/supp-2 Table S1 List of species from the questionnaire and percentage of students (*n* = 485) who correctly named the respective species in pre- and post-test

10.7717/peerj.6581/supp-3 Table S2 Variables used in the statistical models and their justification from available literature

10.7717/peerj.6581/supp-4 Table S3 Number of participants and tests in our study

10.7717/peerj.6581/supp-5 Table S4 Number of students in the final dataset, i.e. students for whom paired pre- and post-tests were analysed for this study

10.7717/peerj.6581/supp-6 Table S5 Correlation of test scores for the different questions Significance (Spearman Rank correlation, *p* < 0.001) indicated in bold. Correlations between naming and assignment tasks are highlighted in grey. *N* = 549.

10.7717/peerj.6581/supp-7 Supplemental Information 1 Dataset of the study

10.7717/peerj.6581/supp-8 Supplemental Information 2 Questionnaire used in the pre-test Original version in German. For copyright reasons the photos depicting the Lamiaceae family in Fig. 1 of the manuscript vary from the photos included in the questionnaire.

10.7717/peerj.6581/supp-9 Supplemental Information 3 Questionnaire used in the post-test The post-test questions are the same as in the pre-test; at the end of the questionnaire questions ask to give personal data about attendance in the course and estimated amount of self-studies. Original version in German. For copyright reasons the photos depicting the Lamiaceae family in Fig. 1 of the manuscript vary from the photos included in the questionnaire.

## Additional Information and Declarations

### Data Availability

The following information was supplied regarding data availability: The raw data are available in the Supplemental Files.
