# Supplementary material for: Just a small bunch of flowers: the botanical knowledge of students and the positive effects of courses in plant identification at German universities
Source: PeerJ. 2019 Mar 13;7:e6581. doi: 10.7717/peerj.6581 (PMC6420800; doi:10.7717/peerj.6581)
Supplement: Table S1 [file peerj-07-6581-s002.docx]

| Species name | German common name(s) | English common name | Plant family | % of students with correct identification in pre/post test | |
| --- | --- | --- | --- | --- | --- |
| *Achillea millefolium* | Wiesen-Schafgarbe | yarrow | Asteraceae | 7.8 | 15.8 |
| *Aegopodium podagraria* | Geißfuß, Girsch | ground-elder | Apiaceae | 1.6 | 9.5 |
| *Ajuga reptans* | Kriechender Günsel | bugle | Lamiaceae | 0.2 | 8 |
| *Alliaria petiolata* | Knoblauchsrauke, Knoblauchskraut, Lauchkraut, Knoblauchhederich | garlic mustard | Brassicaceae | 0.5 | 20.2 |
| *Anemone nemorosa* | Busch-Windröschen | wood anemone | Ranunculaceae | 12.6 | 42.4 |
| *Anthriscus sylvestris* | Wiesen-Kerbel | cow parsley | Apiaceae | 0.5 | 15.1 |
| *Aquilegia vulgaris* | Gewöhnliche Akelei | columbine | Ranunculaceae | 3.5 | 17.3 |
| *Ballota nigra* | Schwarznessel,  Stink-Andorn,  Schwarzer Gottvergess | black horehound | Lamiaceae | 0 | 2.2 |
| *Bellis perennis* | Gänseblümchen | daisy | Asteraceae | 86.2 | 91.1 |
| *Capsella bursa-pastoris* | Hirtentäschel | shepherd's-purse | Brassicaceae | 2.2 | 34.2 |
| *Cardamine pratensis* | Wiesen-Schaumkraut | cuckooflower | Brassicaceae | 2.6 | 14.8 |
| *Centaurea cyanus* | Kornblume | cornflower | Asteraceae | 32.2 | 59 |
| *Cruciata laevipes* | Gewimpertes Kreuzlabkraut | crosswort | Rubiaceae | 0 | 0.2 |
| *Daucus carota* | Wilde Möhre | wild carrot | Apiaceae | 1.1 | 20.9 |
| *Galium odoratum* | Waldmeister,  Wohlriechendes Labkraut | woodruff | Rubiaceae | 5.6 | 26.6 |
| *Galium verum* | Echtes Labkraut | lady's bedstraw | Rubiaceae | 0 | 2.7 |
| *Lamium album* | Weiße Taubnessel | white dead-nettle | Lamiaceae | 2.6 | 43.4 |
| *Lamium galeobdolon*  *Galeobdolon luteum* | Goldnessel,  Gold-Taubnessel | yellow archangel | Lamiaceae | 0.2 | 18.6 |
| *Lotus corniculatus* | Gewöhnlicher Hornklee | common bird's-foot-trefoil | Fabaceae | 0.5 | 17.9 |
| *Lychnis flos-cuculi* | Kuckuck-Lichtnelke | ragged-robin | Caryophyllaceae | 0 | 3.1 |
| *Medicago lupulina* | Hopfenklee, Gelbklee | black medick | Fabaceae | 1.6 | 19.3 |
| *Orlaya grandiflora* | Großblütige Strahlendolde,  Strahlen-Breitsame | large-flowered orlaya | Apiaceae | 0 | 0.2 |
| *Ranunculus acris* | Scharfer Hahnenfuß | meadow buttercup | Ranunculaceae | 0.7 | 26.8 |
| *Ranunculus repens* | Kriechender Hahnenfuß | creeping buttercup | Ranunculaceae | 0.7 | 28.4 |
| *Sherardia arvensis* | Ackerröte | field madder | Rubiaceae | 0 | 0 |
| *Silene dioica* | Rote Lichtnelke | red campion | Caryophyllaceae | 0 | 14.2 |
| *Stellaria holostea* | Große Sternmiere | greater stitchwort | Caryophyllaceae | 0 | 14.2 |
| *Stellaria media* | Vogelmiere | common chickweed | Caryophyllaceae | 0.4 | 11.8 |
| *Taraxacum officinale* | Löwenzahn | dandelion | Asteraceae | 90.9 | 96.4 |
| *Thlaspi arvense* | Acker-Hellerkraut | garlic penny-cress | Brassicaceae | 0 | 3.1 |
| *Trifolium repens* | Weißklee, Kriech-Klee | white clover | Fabaceae | 5.3 | 40.3 |
| *Vicia cracca* | Vogelwicke | tufted vetch | Fabaceae | 0.4 | 17.5 |
