# Supplementary material for: Just a small bunch of flowers: the botanical knowledge of students and the positive effects of courses in plant identification at German universities
Source: PeerJ. 2019 Mar 13;7:e6581. doi: 10.7717/peerj.6581 (PMC6420800; doi:10.7717/peerj.6581)
Supplement: Table S2 [file peerj-07-6581-s003.docx]

| Variable | Explanation, factor levels | Reference(s) | Type |
| --- | --- | --- | --- |
| Model 1 |  | | |
| Score gain | Difference of post-test scores and pre-test scores (sum totals of all points scored in pre- and post-test, respectively) (questions 1, 2a and b, 3a and b) | ‑ | Dependent |
| Test type | Pre-test *or* post-test | ‑ | Explanatory, fixed |
| University/Group/ID |  | ‑ | Random factor |
| Model 2 |  | | |
| Score gain | Difference of post-test scores and pre-test scores (sum totals of all points scored in pre- and post-test, respectively) (questions 1, 2a, 3a) | ‑ | Dependent |
| Knowledge level | Level of knowledge that is required to answer a certain question: ‘taxonomic concept knowledge’ (questions 1 and 2a), ‘declarative species knowledge’ (question 3a) | According to Bloom’s taxonomy of learning (Anderson et al., 2001; Crowe et al., 2008) | Explanatory, fixed |
| Test type | Pre-test or Post-test | ‑ | Explanatory, fixed |
| Test type: knowledge type | Term to test for interaction between the test type and the knowledge type | ‑ | Explanatory, fixed |
| University/Group/ID |  | ‑ | Random factor |
| Model 3 |  | | |
| Score gain | Difference of post-test scores and pre-test scores (sum totals of all points scored in pre- and post-test, respectively) (questions 1, 2a, 3a) |  | Dependent |
| Scores pre-test | Pre-test scores of each learner (sum total of all points scored in the pre-test)  (questions 1, 2a, 3a) | ‑ | Explanatory, fixed |
| Gender | Gender of each learner | [Brähmer (2006)](#_ENREF_2) [Fančovičová and Prokop (2011)](#_ENREF_4) | Explanatory, fixed |
| Latin | Yes/No: Whether or whether not a learner had Latin at school | [Haag (2001)](#_ENREF_5) | Explanatory, fixed |
| Age | Age of participant in years | - | Explanatory, fixed |
| Organisation | Yes/No: Whether or whether not the learner is member of an environmental organization | [Frobel](#_ENREF_10) and Schlumprecht (2016) | Explanatory, fixed |
| Attendance | Percentage of class days on which a participant was present in the course (student´s estimate), from 1 (< 50 %) to 2 (> 50 %) | [Bromme](#_ENREF_5) et al. (2004) | Explanatory, fixed |
| Self-study | Hours spent on self-study (student´s estimate), from 1 (up to 1 h self-study per week) to 2 (more than 1 hour of self-study per week) | - | Explanatory, fixed |
| University/Group |  | ‑ | Random factor |
| Model 4 |  |  |  |
| Score gain | Difference of post-test scores and pre-test scores (sum totals of all points scored in pre- and post-test, respectively) (questions 1, 2a, 3a) | ‑ | Dependent |
| Group size | Minimal group size of a learning group, equals the number of returned paired pre- and post-tests | [Bromme](#_ENREF_5) et al. (2004) | Explanatory, fixed |
| Field trip | Yes/No, Whether or whether not field trip (teaching and learning outside of the classroom) were part of the course | Rickingson et al (2004) Fančovičová and Prokop (2011) | Explanatory, fixed |
| Study programme | Study programme in which a student was enrolled | - | Explanatory, fixed |
| Instructor | Instructor for the respective course(s) | [Hattie (2009)](#_ENREF_6) | Explanatory, fixed |
| University |  |  | Random factor |

**References**

Anderson, L. W., Krathwohl, D. R., & Bloom, B. S. (2001). *A taxonomy for learning, teaching, and assessing: A revision of Bloom's taxonomy of educational objectives*: Allyn & Bacon.

Brähmer, R. (2006). *Natur obskur: wie Jugendliche huete die Natur erfahren*. München: Oekom.

Crowe, A., Dirks, C., & Wenderoth, M. P. (2008). Biology in bloom: implementing Bloom's taxonomy to enhance student learning in biology. *CBE-Life Sciences Education, 7*(4), 368-381.

Fančovičová, J., & Prokop, P. (2011). Plants have a chance: outdoor educational programmes alter students' knowledge and attitudes towards plants. *Environmental Education Research, 17*(4), 537-551.

Haag, L. (2001). Auswirkungen von Lateinunterricht-Ergebnisse nach zwei Lernjahren. *Psychologie in Erziehung und Unterricht, 48*, 30-37.

Hattie, J. A. C. (2009). *Visible Learning. A synthesis of over 800 meta-analyses relating to achievement.* London & New York: Routledge.
