# Supplementary material for: Just a small bunch of flowers: the botanical knowledge of students and the positive effects of courses in plant identification at German universities
Source: PeerJ. 2019 Mar 13;7:e6581. doi: 10.7717/peerj.6581 (PMC6420800; doi:10.7717/peerj.6581)
Supplement: Table S3 [file peerj-07-6581-s004.docx]

| University | Total no. of students | No. of pre-tests | No. of post-tests | No. of paired tests |
| --- | --- | --- | --- | --- |
| Bremen | 207 | 170 (82.1%) | 84 (40.6%) | 45 (21.7%) |
| Flensburg | 75 | 60 (80.0%) | 52 (69.3%) | 45 (60.0%) |
| Frankfurt | 58 | 58 (100%) | 51 (87.9%) | 51 (87.9%) |
| Gießen | 280 | 280 (100%) | 132 (47.1%) | 132 (47.1%) |
| Hannover | 65 | 44 (67.7%) | 38 (58.5%) | 17 (26.2%) |
| Kassel | 106 | 104 (98.1%) | 86 (81.1%) | 83 (78.3%) |
| Lüneburg | 110 | 100 (90.1%) | 66 (60.0%) | 56 (50.9%) |
| Mainz | 159 | 150 (94.3%) | 132 83.0%) | 120 (75.5%) |
| Total | 1060 | 966 (91.1%) | 640 (60.4%) | 549 (51.8%) |
