# Supplementary material for: Just a small bunch of flowers: the botanical knowledge of students and the positive effects of courses in plant identification at German universities
Source: PeerJ. 2019 Mar 13;7:e6581. doi: 10.7717/peerj.6581 (PMC6420800; doi:10.7717/peerj.6581)
Supplement: Table S4 [file peerj-07-6581-s005.docx]

| University | Study programme(s) | No. of students | Age  median range | Gender ratio % male | Field trip | No. of groups | Group size range |
| --- | --- | --- | --- | --- | --- | --- | --- |
| Bremen | Teaching Biology, BA Biology, BSc. other | 16 19 10 | 21 17-29 | 31 | No | 2 | 17-28 |
| Flensburg | Teaching Biology, BA | 45 | 21 19-32 | 13 | Yes | 2 | 17-24 |
| Frankfurt | Teaching Biology, BA/Staatsexamen Biology, BSc. | 35 16 | 20 18-29 | 35 | No | 2 | 25-26 |
| Gießen | Teaching Biology, Staatsexamen Biology, BSc. | 95 37 | 21 18-29 | 30 | (Yes) (50% of the students) | 3 | 28-65 |
| Hannover | Teaching Biology Biology, BSc. other | 4 10 3 | 19.5 19-26 | 29 | Yes | 1 | 17 |
| Kassel | Teaching Biology, BA/Staatsexamen  Biology, BSc. or Diploma  other | 38 44 1 | 21 19-32 | 31 | Yes | 3 | 24-31 |
| Lüneburg | Teaching Biology, BA Environmental Sciences, BSc. | 28 28 | 22 19-35 | 21 | No | 3 | 18-19 |
| Mainz | Teaching Biology, BA  Biology, BSc. or Diploma  other | 32 84 4 | 21 19-29 | 30 | Yes | 4 | 23-37 |
| Total |  | 549 | 21 17-35 | 28.4 |  | 20 | 17-65 |
