# Supplementary material for: Just a small bunch of flowers: the botanical knowledge of students and the positive effects of courses in plant identification at German universities
Source: PeerJ. 2019 Mar 13;7:e6581. doi: 10.7717/peerj.6581 (PMC6420800; doi:10.7717/peerj.6581)
Supplement: Table S5 — Significance (Spearman Rank correlation, p < 0.001) indicated in bold. Correlations between naming and assignment tasks are highlighted in grey. N = 549. [file peerj-07-6581-s006.docx]

| Question | question 3a | question 3b | question 2a | question 2b | question 1 |
| --- | --- | --- | --- | --- | --- |
| Task | naming species | assignment species | naming character | assignment character | naming family |
| Pre-tests |  |  |  |  |  |
| naming species | **1.000** |  |  |  |  |
| assignment species | **0.970** | **1.000** |  |  |  |
| naming character | 0.073 | 0.073 | **1.000** |  |  |
| assignment character | 0.066 | 0.076 | **0.964** | **1.000** |  |
| naming family | **0.235** | **0.223** | **0.431** | **0.436** | **1.000** |
| Post-tests |  |  |  |  |  |
| naming species | **1.000** |  |  |  |  |
| assignment species | **0.972** | **1.000** |  |  |  |
| naming character | **0.256** | **0.265** | **1.000** |  |  |
| assignment character | **0.256** | **0.285** | **0.970** | **1.000** |  |
| naming family | **0.533** | **0.527** | **0.567** | **0.558** | **1.000** |
| Test gain |  |  |  |  |  |
| naming species | **1.000** |  |  |  |  |
| assignment species | **0.959** | **1.000** |  |  |  |
| naming character | **0.216** | **0.235** | **1.000** |  |  |
| assignment character | **0.213** | **0.242** | **0.971** | **1.000** |  |
| naming family | **0.403** | **0.394** | **0.433** | **0.433** | **1.000** |
