# Supplementary material for: Just a small bunch of flowers: the botanical knowledge of students and the positive effects of courses in plant identification at German universities
Source: PeerJ. 2019 Mar 13;7:e6581. doi: 10.7717/peerj.6581 (PMC6420800; doi:10.7717/peerj.6581)
Supplement: Supplemental Information 2 — Original version in German. For copyright reasons the photos depicting the Lamiaceae family in Fig. 1 of the manuscript vary from the photos included in the questionnaire. [file peerj-07-6581-s008.pdf]

# Pretest 2013

Visuelles Zuordnungsverfahren

## Biodiversität im Kontext universitärer Lehrerbildung

### Das Visuelle Zuordnungsverfahren

Fragebogen zu taxonomischen Kenntnissen

**Bitte füllen Sie folgende Felder vollständig aus:**

Datum:

Hochschule:

Dozent:

Persönliche ID:

| 1 | 2 | 3 | 4 | 5 |
|---|---|---|---|---|
| | | | | |

**Ihre Persönliche ID setzt sich aus den Anfangsbuchstaben folgender individueller Angaben in der genannten Reihenfolge zusammen:**

1) Erster Buchstabe des Geburtsorts
2) Zweiter Buchstabe des Vornamens
3) Dritter Buchstabe des Nachnamens
4) Zweite Ziffer des Geburtstags
5) Zweite Ziffer des Geburtsmonats

**Beispiel:** Die aus **B**erlin stammende J**u**lia Mü**l**ler, geboren am 1**3**.0**7**.1988 hat die ID: **BUL37**

# Botanik

Auswertung

**1) Nennen Sie die Familie, unter der sich die abgebildeten Organismen zusammenfassen lassen.**

| |
|---|
| |

**2) Nennen Sie allgemeine Merkmale der abgebildeten Familie und belegen Sie diese mit Abbildungen.**

| Merkmale | Abbildungen |
|---|---|
| | |
| | |
| | |

**3) Nennen Sie die abgebildeten Arten und belegen Sie diese mit Abbildungen.**

| Arten | Abbildungen |
|---|---|
| | |
| | |
| | |
| | |

**4) Nennen Sie die Familie, unter der sich die abgebildeten Organismen zusammenfassen lassen.**

| |
|---|
| |

**5) Nennen Sie allgemeine Merkmale der abgebildeten Familie und belegen Sie diese mit Abbildungen.**

| Merkmale | Abbildungen |
|---|---|
| | |
| | |
| | |

**6) Nennen Sie die abgebildeten Arten und belegen Sie diese mit Abbildungen.**

| Arten | Abbildungen |
|---|---|
| | |
| | |
| | |
| | |

**7) Nennen Sie die Familie, unter der sich die abgebildeten Organismen zusammenfassen lassen.**

| |
|---|
| |

**8) Nennen Sie allgemeine Merkmale der abgebildeten Familie und belegen Sie diese mit Abbildungen.**

| Merkmale | Abbildungen |
|---|---|
| | |
| | |
| | |

**9) Nennen Sie die abgebildeten Arten und belegen Sie diese mit Abbildungen.**

| Arten | Abbildungen |
|---|---|
| | |
| | |
| | |
| | |

**10) Nennen Sie die Familie, unter der sich die abgebildeten Organismen zusammenfassen lassen.**

| |
|---|
| |

**11) Nennen Sie allgemeine Merkmale der abgebildeten Familie und belegen Sie diese mit Abbildungen.**

| Merkmale | Abbildungen |
|---|---|
| | |
| | |
| | |

**12) Nennen Sie die abgebildeten Arten und belegen Sie diese mit Abbildungen.**

| Arten | Abbildungen |
|---|---|
| | |
| | |
| | |
| | |

**13) Nennen Sie die Familie, unter der sich die abgebildeten Organismen zusammenfassen lassen.**

**14) Nennen Sie allgemeine Merkmale der abgebildeten Familie und belegen Sie diese mit Abbildungen.**

| Merkmale | Abbildungen |
|---|---|
| | |
| | |
| | |

**15) Nennen Sie die abgebildeten Arten und belegen Sie diese mit Abbildungen.**

| Arten | Abbildungen |
|---|---|
| | |
| | |
| | |
| | |

**16) Nennen Sie die Familie, unter der sich die abgebildeten Organismen zusammenfassen lassen.**

**17) Nennen Sie allgemeine Merkmale der abgebildeten Familie und belegen Sie diese mit Abbildungen.**

| Merkmale | Abbildungen |
|---|---|
| | |
| | |
| | |

**18) Nennen Sie die abgebildeten Arten und belegen Sie diese mit Abbildungen.**

| Arten | Abbildungen |
|---|---|
| | |
| | |
| | |
| | |

**19) Nennen Sie die Familie, unter der sich die abgebildeten Organismen zusammenfassen lassen.**

**20) Nennen Sie allgemeine Merkmale der abgebildeten Familie und belegen Sie diese mit Abbildungen.**

| Merkmale | Abbildungen |
|---|---|
| | |
| | |
| | |

**21) Nennen Sie die abgebildeten Arten und belegen Sie diese mit Abbildungen.**

| Arten | Abbildungen |
|---|---|
| | |
| | |
| | |
| | |

**22) Nennen Sie die Familie, unter der sich die abgebildeten Organismen zusammenfassen lassen.**

**23) Nennen Sie allgemeine Merkmale der abgebildeten Familie und belegen Sie diese mit Abbildungen.**

| Merkmale | Abbildungen |
|---|---|
| Spaltfrucht | |
| Trugdolde | |
| Scheinquirl (Stipeln sind wie Laubblätter gestaltet) | |

**24) Nennen Sie die abgebildeten Arten und belegen Sie diese mit Abbildungen.**

| Arten | Abbildungen |
|---|---|
| Galium odoratum (Waldmeister) | |
| Cruciata laevipes (Gewimpertes Kreuzlabkraut) | |
| Galium verum (Echtes Labkraut) | |
| Sherardia arvensis (Ackerröte) | |

## Persönliche Daten

**A) Bitte geben Sie die Art Ihres Studiengangs an.**

Diplom Biologie ☐ B.A. Biologie ☐ B.A. Lehramt ☐ Andere: ______

**B) Bitte geben Sie Ihr Geschlecht an.**

weiblich ☐ männlich ☐

**C) Bitte geben Sie Ihr Alter an.**

______ Jahre

**D) Wie viele Einwohner hatte der Ort, in dem Sie bis zum 12. Lebensjahr gewohnt haben?**

< 10.000 ☐ 10.000 – 30.000 ☐ 30.000 – 60.000 ☐ > 60.000 ☐

**E) Wie lässt sich die direkte Umgebung, in der Sie in dieser Zeit vorwiegend gewohnt haben, am besten charakterisieren?**

ländlich ☐ ☐ ☐ ☐ ☐ städtisch

**F) Wie viele Einwohner hatte der Ort, in dem Sie vom 12. bis zum 18. Lebensjahr gewohnt haben?**

< 10.000 ☐ 10.000 – 30.000 ☐ 30.000 – 60.000 ☐ > 60.000 ☐

**G) Wie lässt sich die direkte Umgebung, in der Sie in dieser Zeit vorwiegend gewohnt haben, am besten charakterisieren?**

ländlich ☐ ☐ ☐ ☐ ☐ städtisch

**H) Hatten Sie in der Schule das Fach Latein?**

Nein ☐ Ja ☐

**I) Sind oder waren Sie Mitglied in einem oder mehreren naturverbundenen Organisationen (z.B. Pfadfinder, BUND, etc.)?**

Nein ☐ Ja, in folgenden: ______
