# Supplementary material for: Just a small bunch of flowers: the botanical knowledge of students and the positive effects of courses in plant identification at German universities
Source: PeerJ. 2019 Mar 13;7:e6581. doi: 10.7717/peerj.6581 (PMC6420800; doi:10.7717/peerj.6581)
Supplement: Supplemental Information 3 — The post-test questions are the same as in the pre-test; at the end of the questionnaire questions ask to give personal data about attendance in the course and estimated amount of self-studies. Original version in German. For copyright reasons the photos depicting the Lamiaceae family in Fig. 1 of the manuscript vary from the photos included in the questionnaire. [file peerj-07-6581-s009.pdf]

Posttest 2013

VZ
Visuelles Zuordnungsverfahren

Biodiversität im Kontext universitärer Lehrerbildung

Das Visuelle Zuordnungsverfahren

Fragebogen zu taxonomischen Kenntnissen

Bitte füllen Sie folgende Felder vollständig aus:

Datum:	
Hochschule:	
Dozent:	
Persönliche ID:	1 | 2 | 3 | 4 | 5

Ihre Persönliche ID setzt sich aus den Anfangsbuchstaben folgender individueller Angaben in der genannten Reihenfolge zusammen:

1) Erster Buchstabe des Geburtsorts
2) Zweiter Buchstabe des Vornamens
3) Dritter Buchstabe des Nachnamens
4) Zweite Ziffer des Geburtstags
5) Zweite Ziffer des Geburtsmonats

Beispiel: Die aus **B**erlin stammende J**u**lia Mü**l**ler, geboren am 1**3**.0**7**.1988 hat die ID: **BUL37**

Botanik

Auswertung

1) Nennen Sie die Familie, unter der sich die abgebildeten Organismen zusammenfassen lassen.

2) Nennen Sie allgemeine Merkmale der abgebildeten Familie und belegen Sie diese mit Abbildungen.

Merkmale	Abbildungen

3) Nennen Sie die abgebildeten Arten und belegen Sie diese mit Abbildungen.

Arten	Abbildungen

4) Nennen Sie die Familie, unter der sich die abgebildeten Organismen zusammenfassen lassen.

5) Nennen Sie allgemeine Merkmale der abgebildeten Familie und belegen Sie diese mit Abbildungen.

Merkmale	Abbildungen

6) Nennen Sie die abgebildeten Arten und belegen Sie diese mit Abbildungen.

Arten	Abbildungen

7) Nennen Sie die Familie, unter der sich die abgebildeten Organismen zusammenfassen lassen.

8) Nennen Sie allgemeine Merkmale der abgebildeten Familie und belegen Sie diese mit Abbildungen.

Merkmale	Abbildungen

9) Nennen Sie die abgebildeten Arten und belegen Sie diese mit Abbildungen.

Arten	Abbildungen

10) Nennen Sie die Familie, unter der sich die abgebildeten Organismen zusammenfassen lassen.

11) Nennen Sie allgemeine Merkmale der abgebildeten Familie und belegen Sie diese mit Abbildungen.

Merkmale	Abbildungen

12) Nennen Sie die abgebildeten Arten und belegen Sie diese mit Abbildungen.

Arten	Abbildungen

13) Nennen Sie die Familie, unter der sich die abgebildeten Organismen zusammenfassen lassen.

14) Nennen Sie allgemeine Merkmale der abgebildeten Familie und belegen Sie diese mit Abbildungen.

Merkmale	Abbildungen

15) Nennen Sie die abgebildeten Arten und belegen Sie diese mit Abbildungen.

Arten	Abbildungen

16) Nennen Sie die Familie, unter der sich die abgebildeten Organismen zusammenfassen lassen.

17) Nennen Sie allgemeine Merkmale der abgebildeten Familie und belegen Sie diese mit Abbildungen.

Merkmale	Abbildungen

18) Nennen Sie die abgebildeten Arten und belegen Sie diese mit Abbildungen.

Arten	Abbildungen

19) Nennen Sie die Familie, unter der sich die abgebildeten Organismen zusammenfassen lassen.

20) Nennen Sie allgemeine Merkmale der abgebildeten Familie und belegen Sie diese mit Abbildungen.

Merkmale	Abbildungen

21) Nennen Sie die abgebildeten Arten und belegen Sie diese mit Abbildungen.

Arten	Abbildungen

22) Nennen Sie die Familie, unter der sich die abgebildeten Organismen zusammenfassen lassen.

23) Nennen Sie allgemeine Merkmale der abgebildeten Familie und belegen Sie diese mit Abbildungen.

Merkmale	Abbildungen
Spaltfrucht	
Trugdolde	
Scheinquirl (Stipeln sind wie Laubblätter gestaltet)	

24) Nennen Sie die abgebildeten Arten und belegen Sie diese mit Abbildungen.

Arten	Abbildungen
Galium odoratum (Waldmeister)	
Cruciata laevipes (Gewimpertes Kreuzlabkraut)	
Galium verum (Echtes Labkraut)	
Sherardia arvensis (Ackerröte)	

Persönliche Daten

J) Wie oft (in Prozent) waren Sie in diesem Semester an den taxonomischen Veranstaltungen anwesend?

☐ 5-25% ☐ 26-50% ☐ 51-75% ☐ 76-100%

K) Wie viele Stunden haben Sie wöchentlich mit dem Selbststudium verbracht?

☐ 0 Stunden ☐ 1 Stunde ☐ 2 Stunden ☐ 3 Stunden ☐ Mehr als 3 Stunden

L) Zu welcher dieser Fachrichtungen haben sie dieses Semester Veranstaltungen besucht?

☐ Botanik ☐ Zoologie

M) Geben Sie bitte den Namen der Dozenten an, bei denen sie die folgenden Veranstaltungen besucht haben (Nicht besuchte bzw. nicht angebotene Veranstaltungen leer lassen)

	Botanik
Exkursion	
Praktikum/Übung	
Vorlesung	
Andere	

N) Die Methode der Vermittlung botanischer Inhalte der Arten- und Formenkenntnis war interessant

trifft nicht zu -- ☐ ------------- ☐ ------------- ☐ ------------- ☐ ------------- ☐ -- **trifft in vollem Umfang zu**

O) Die besuchten Veranstaltungen haben mir einen guten Überblick über botanische Arten und Formen vermittelt

trifft nicht zu -- ☐ ------------- ☐ ------------- ☐ ------------- ☐ ------------- ☐ -- **trifft in vollem Umfang zu**

P) Die besuchten Veranstaltungen haben mein persönliches Interesse an der botanischen Arten- und Formenkenntnis geweckt oder gesteigert

trifft nicht zu -- ☐ ------------- ☐ ------------- ☐ ------------- ☐ ------------- ☐ -- **trifft in vollem Umfang zu**

Vielen Dank für Ihre Teilnahme!
